# Childhood sexual abuse among Black men who have sex with men: A cornerstone of a syndemic?

**DOI:** 10.1371/journal.pone.0206746

**Published:** 2018-11-01

**Authors:** Elwin Wu

**Affiliations:** Social Intervention Group, Columbia University School of Social Work, New York, NY, United States of America; International AIDS Vaccine Initiative, UNITED STATES

## Abstract

**Background:**

The sequelae of childhood sexual abuse (CSA) includes HIV infection, engagement in HIV risk behaviors, substance misuse, and intimate partner violence (IPV). Although Black men who have sex with men (MSM) are disproportionately infected with HIV in the U.S.—especially in urban locations such as New York City—there is limited research with larger samples of Black MSM of varied HIV status regarding the prevalence of CSA and the potential negative consequence with respect to a “syndemic,” i.e., the co-occurrence of adverse conditions such as HIVrisk, substance misuse, and IPV.

**Methods:**

Black MSM (*N* = 1,002) recruited in New York City from 2009–2015 completed a screening assessment eliciting self-reported data on age, CSA, self-reported HIV status, number of male sexual partners, number of acts of condomless anal intercourse (CAI), substance misuse, and IPV. Hypothesis testing utilized logistic and linear regression models with self-reported data on CSA (independent variable) and indicators of the following syndemic factors: HIV risk, substance misuse, and IPV.

**Results:**

More than one-fourth (28.1%) met criteria for experiencing CSA. CSA was associated with significantly greater odds of being HIV-positive (*AOR* = 1.5; *95% CI* = 1.1–2.0); number of male sexual partners (*b* = 2.0, *SE* = 0.5, *p* = .002) and condomless acts of anal intercourse (*b* = 4.3, *SE* = 1.6, *p* = .007); odds of binge drinking (*AOR* = 1.5; *95% CI* = 1.1–2.0) and illicit substance use (*AOR* = 1.5; *95% CI* = 1.1–2.0); and odds of experiencing current IPV (*AOR* = 1.7; *95% CI* = 1.2–2.3). CSA was associated with significantly greater odds of concurrently experiencing 2 or more syndemic factors (*AOR* = 2.0, 95% *CI* = 1.4–2.9, *p* < .001); concurrently experiencing 2 or more syndemic factors was significantly associated with having a riskier HIV status (for being HIV-positive: *AOR* = 1.5, 95% *CI* = 1.1–2.1, *p* = .02; for having an unknown HIV status: *AOR* = 3.7, 95% *CI* = 1.9–12.9, *p* = .04).

**Conclusions:**

Among Black MSM, CSA is a prevalent problem and is a significant antecedent to HIV, substance misuse, and IPV indicators and risk. Addressing CSA may be a valuable approach to remedy the syndemic of HIV, substance misuse, and violence that has burdened MSM, especially Black MSM, in the U.S.

## Introduction

Men who have sex with men (MSM) have a disproportionate burden of the HIV/AIDS epidemic since its onset in the U.S. [1,2]. The majority of annual HIV transmissions in the U.S. remains via male-to-male sexual contact [1]. MSM have almost a four-fold higher lifetime risk of becoming infected by HIV compared to the next highest risk group (women who inject drugs) [1]. Since 2008, the number of annual HIV diagnoses among Black MSM has exceeded those among White MSM [3] despite that the size of the White non-Hispanic population size is 6 times that of the Black population in the U.S. The disparity experienced is exemplified by current estimates of risk for HIV acquisition [3]: while Hispanic and White MSM have elevated lifetime risk of HIV diagnoses compared to other populations (1 in 4 and 1 in 11 respectively), Black MSM have a 1 in 2 lifetime risk of becoming infected by HIV. Based on current incidence rates, one model predicted that more than 60% of Black MSM in the U.S. would be infected with HIV by age 40 [4].

HIV health disparities experienced by MSM have proven to be persistent despite the successes in biomedical and behavioral prevention and treatment efforts to date. Advances such as antiretroviral therapy that can achieve viral suppression, pre-exposure prophylaxis (PrEP), behavioral risk reduction programs, and structural interventions (e.g., needle and syringe programs) that have resulted in decreases since 2000 in transmissions from mother-to-child, injecting drug use, and heterosexual transmission; yet, MSM have witnessed an increase in transmission rates over the same time period [2,3]. The entrenched disparity of HIV among MSM has been posited to arise out of a “syndemic,” the co-occurrence of multiple health and psychsocial problems that interact, generally reinforcing, each other [5]. The hallmark of a syndemic is an increase in the likelihood for experiencing 2 or more health and psychosocial problems concurrently compared to none or a single problem. For MSM, the origins and great deal of research regarding theory of syndemics applied to MSM involve HIV, substance misuse, mental health, and more recently, intimate partner violence (IPV) [6,7,8,9,10,11,12,13].

Roots of the syndemic among MSM have been posited to lie in early experiences of stress and stigma, which in turn increase the likelihood of mental health problems, substance misuse, sexual risk behavior, and difficulty forming/maintaining healthy intimate relationships (or dissolving unhealthy intimate relationships), all of which in turn increase or exacerbate the presence of the other components of the syndemic [14]. Childhood sexual abuse (CSA) is not only a developmentally early stressor, much research has documented the link to subsequent problems like HIV, substance misuse, mental health problems, and IPV among many populations [15,16,17]. CSA has also garnered attention as a precursor to increased risk for HIV, especially given some evidence of elevated rates of experiencing CSA among MSM (for a review, see Llyod & Operario [18] and Schafer *et al*. [19]). Some studies focused on the syndemic among MSM have also suggested that CSA may be a salient factor to consider [6,9,20]. None of these studies specifically focused on prevalence of CSA among Black MSM, and though they may have reported some estimates, they are limited by small sample sizes for Black MSM. There has been one published one multi-site study examining CSA among Black MSM, but the sample size in any one of the cities was capped at about 225 individuals; moreover, the sampling plan weighted very heavily (~20:1) towards Black MSM who believed they were not living with HIV; that is, Black MSM living with HIV were heavily undersampled, making it unclear how generalizable the observed associations between CSA and syndemic factors may be to the larger population of Black MSM that has a high prevalence of HIV. Consequently, using a relatively large (*N* = 1,002) sample of Black MSM from the New York City area—where HIV prevalence rates among Black MSM have been estimated to exceed HIV rates in many countries in Sub-Saharan Africa [21]—recruited without regard to perceived HIV status, this study addresses the following research questions:

- What is the prevalence of CSA among this sample of black MSM?
- Is the experience of CSA associated with other syndemic factors—HIV risk indicators, substance misuse, and experiencing IPV—among this sample of black MSM?
- Is the experience of CSA associated with the presence of a syndemic—operationalized as the co-occurrence of more than one of the following: engaging in HIV risk behavior, substance misuse, and experiencing IPV—among this sample of black MSM?

## Materials and methods

### Study design and sample

This study utilized data obtained during the screening portion of a randomized clinical trial testing a couple-based, behavioral preventive intervention for black MSM couples at elevated risk for HIV/STI transmission. The parent study sought to enroll a sample of black MSM in same sex relationships where one or both partners engaged in illicit use of drugs and engaged in sexual risk behavior(s). Recruitment was conducted from November 2009 to November 2015 at local service agencies, bars, clubs, and community events frequented by MSM in the New York City area; the study also recruited using the internet (e.g., study website, Facebook) and social media apps (e.g., Grindr). Potential participants screened for inclusion in the parent study were informed that the intervention consisted of an orientation session followed by 4 weekly sessions in which both partners conjointly worked with a facilitator to address threats (e.g., HIV, substance misuse) to the well-being, physical health, and sexual health of black men in same-sex relationships.

For this secondary analysis of screening data, all cases were included from the screening database for which the respondent met the following criteria to be considered a black MSM for the purposes of the study/analyses reported herein: (1a) identify as male currently; (1b) assigned male gender at birth; (2) identify as Black and/or African American; (3) had non-coerced sex with another man during the prior 3 months; and (4) was ≥18 years old. Cases for which the respondent did not provide self-reported information to ascertain CSA (*n* = 11) were excluded from the analyses. The final sample size for this study was 1,002.

The Columbia University Institutional Review Board (Morningside IRB) approved all protocols, materials, and information used in this study, including waiving the requirement of documenting written informed consent as too burdensome given the brief and low risk nature of the screening interview and documenting verbal consent for screening via written attestation by the Research Assistance conducting the screening interview.

### Measures

The screening questionnaire included items to prompt participants to self-report very basic sociodemographic information necessary to establish eligibility for the parent study (i.e., including age, race/ethnicity). All other measures used in this study were derived from multiple choice or close-ended questions.

CSA was assessed by asking whether the respondent has sex or sexual contact before the age of 17. If the respondent indicated yes, then the respondent was coded as reporting CSA if he indicated yes to any of the following questions: (1) Did the sexual contact involve force or coercion? (2) Was the other person 4 or more years older than the respondent? (3) Was the respondent less than 11 years old at the time.

HIV risk indicators included self-reported HIV status (positive, negative, unknown), number of male sexual partners in the past 90 days, and number of acts of condomless anal intercourse (CAI) in the past 90 days. To minimize the potential impact of outliers, respondents who reported more than 30 male partners and 90 acts of CAI in the past 90 days were rectified to 30 partners and 90 acts respectively.

Substance misuse was assessed using an abbreviated version of the drug use portion of the National Institute on Drug Abuse Risk Behavior Assessment [22]. Thus, we screen for the use of the following substances: binge drinking (operationalized as “five or more drinks in a single period”), marijuana, powdered cocaine, rock/crack cocaine, heroin, methamphetamine, and other “party drugs” (e.g., ketamine/“special K”, ecstasy/“X”). Current substance misuse was coded as Yes if use was reported to occur in the past 90 days.

IPV was assessed using the Revised Conflict Tactics Scale (CTS2) [23] (Note: the phrase “not part of BDSM/role play” was added to the sexual IPV items). Items related to minor psychological violence (e.g., “shouted at partner,” “stomped out of room”) were not included due to concerns about low specificity with respect to IPV. For this study, the CTS2 was supplemented with questions involving “threatening to out” and “actually outing” a respondent’s sexuality/sex with men behavior—herein referred to as “gay-related” IPV—and “threatening to disclose” and “actually disclosing” a respondent as HIV-positive (regardless of actual status)—herein referred to as “HIV-related” IPV—using the same answer choices as other CTS2 questions. The reliability of the CTS2 was assessed for the set of items falling within each of the CTS2 categories (i.e., psychological, physical, sexual, and injurious) using Cronbach’s α; results from this sample of predominantly HIV-positive, black MSM ranged from .72 - .81, which is comparable to those reported by the originators of the CTS2 [23]. In this manuscript focused on the current experience of IPV, responses were dichotomized, with any report of experiencing within the past 30 days within a category resulted in coding Yes for currently experiencing that category/type of IPV; if no responses indicated that the experience occurred ever nor within the past 30 days, the responded was coded as No for currently experiencing that category/type of IPV.

To test hypotheses about the co-occurrence of syndemic factors, the following algorithm was employed to “count” the number of syndemic factors occurring with a participant in an analogous fashion to other investigations of a syndemic among MSM [6,9]. The presence of engaging in HIV risk behavior was coded by employing the Boolean OR operator with the following: Did the participant report currently having more than 1 male sexual partner in the past 90 days? and Did the participant report engaging in CAI in the past 90 days? The presence of engaging in substance misuse was coded by employing the Boolean OR operator with the following: Did the participant report binge drinking in the past 90 days? and Did the participant report illicit use of any other substance in the past 90 days? The presence of experiencing IPV remained coded as noted above. All of these variable were thus binary (1 = presence of the particular syndemic factor; 0 = absence of the particular syndemic factor) for a participant. Finally, a sum of the number of these three syndemic factors that was calculated for each participant, which allows for inference about the presence of more than 1 syndemic factor for that participant.

### Analyses

Statistical analyses were performed using SPSS Version 21. To test whether CSA is associated with syndemic factors, multivariate regression was used to assess test hypothesis regarding the focal independent variable of CSA with each of the measures of the following syndemic factors as a dependent variable: HIV risk indicators, substance misuse, and experience of IPV. All multivariate models controlled for age; for all outcome measures other than HIV status, covariance adjustment included HIV status (dummy coded with HIV-negative as the reference group). For HIV status as the dependent variable, multinomial logistic regression was employed using HIV-negative as the reference group. For binary dependent variables, logistic regression was employed. For ratio-level dependent variables, linear regression was employed. Inferential hypothesis testing relied on the regression parameter estimates for CSA (i.e., unstandardized coefficient *b* for linear regression, odds ratios [ORs] for logistic regression), their associated standard errors, and corresponding *p*-values. The criterion level used to determine significance was *p* < .05.

## Results

### Characteristics of the sample

The mean age of the sample was 35.8 years (*SD* = 11.6). Distribution of the various syndemic factors among the sample are presented in Table 1. With respect to substance misuse, 755 (75.3%) respondents reported current illicit use of substances. With respect to IPV, 229 (22.9%) respondent met criteria for currently experiencing IPV according to CTS2 criteria; if we include the additional gay- and HIV-related IPV items, 235 (23.5%) reported currently experiencing some form of IPV.

**Table 1:** Syndemic-related characteristics of the sample of Black MSM (*N* = 1,002).

HIV Status	
HIV-negative	*n* = 366 (36.5%)
HIV-positive	*n* = 595 (59.4%)
Unknown	*n* = 41 (4.1%)
# of acts of CAI (past 90 days)	$\bar{x}$ = 14.8 (*SD* = 22.9)
# of male sexual partners (past 90 days)	$\bar{x}$ = 4.5 (*SD* = 7.2)
Substance misuse (past 90 days)	
Binge drinking	*n* = 401 (40.0%)
Marijuana	*n* = 649 (64.8%)
Cocaine	*n* = 329 (32.8%)
Methamphetamine	*n* = 137 (13.7%)
Heroin	*n* = 33 (3.3%)
Party drug(s)	*n* = 241 (24.1%)
Any illicit substance use	*n* = 755 (75.3%)
Experienced intimate partner violence (past 30 days)	
Psychological (severe)	*n* = 166 (16.6%)
Physical	*n* = 104 (10.4%)
Sexual	*n* = 61 (6.1%)
Injurious	*n* = 48 (4.8%)
Gay-related	*n* = 52 (5.2%)
HIV-related	*n* = 18 (1.8%)
Any intimate partner violence	*n* = 235 (23.5%)

About three-fourths (*n* = 767, 76.5%) reported sexual contact before the age of 17. Among those, 242 (31.5% of those reporting sexual contact before the age of 17) reported experiencing force/coercion during some of these episodes, 250 (32.5%) reported the other person(s) being 4 or more years older, and 166 (21.6%) reported sexual contact before the age of 11. Among the entire sample, more than a quarter (*n* = 282, 28.1%) met criteria for CSA.

### Syndemic factors and childhood sexual abuse

**Fig 1:**
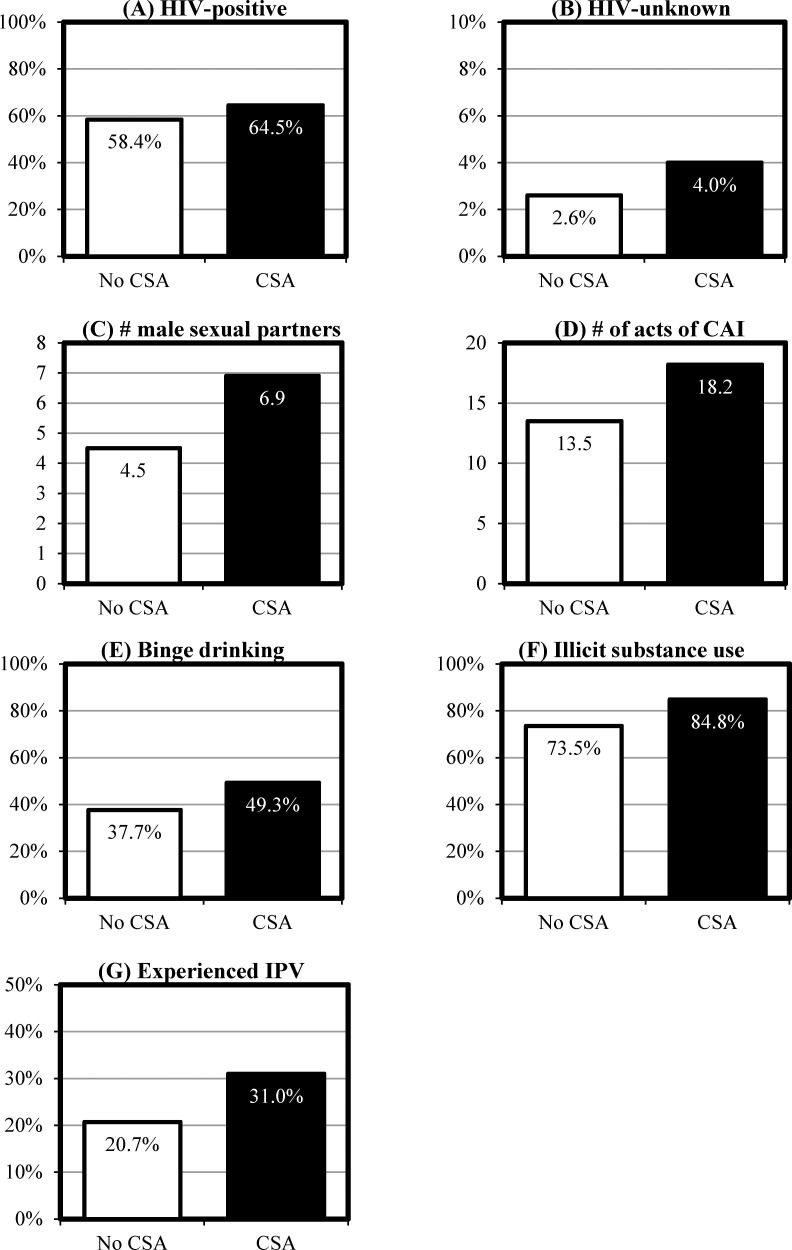
Prevalence of syndemic factors by childhood sexual abuse among the sample of black MSM (*N* = 1,002). (A) HIV-positive. (B) HIV-unknown. (C) Number of male sexual partners in past 90 days. (D) Number of acts of CAI in past 90 days. (E) Engaged in binge drinking in past 90 days. (F) Engaged in illicit use of substances in the past 90 days. (G) Experienced IPV in past 30 days.

Fig 1 depicts the prevalence of various syndemic-related measures, separated by those who did not report CSA (*n* = 720) and those whose self-report indicated that they met the criteria for CSA (*n* = 282).

#### Hypothesis testing

Table 2 summarizes the findings from multivariate models examining the association between CSA and each of the syndemic factor measures. CSA was significantly associated with greater likelihood of being HIV-positive vs. HIV-negative after controlling for age; while not knowing one’s HIV status (vs. HIV-negative) was suggestive (*AOR* = 2.0, 95% *CI* = 1.0–1.1, *p* = .10), we could not rule out the null hypothesis based on an a priori criterion level of α < .05. CSA was significantly associated with greater risks for all of the other syndemic factors assessed (binge drinking, number of male partners, number of acts of CAI, illicit use of substances, and IPV).

**Table 2:** Association between childhood sexual abuse and syndemic factors among the sample of black MSM (*N* = 1,002).

	HIV-positive^a^	HIV-unknown^a^	# of male sexual partners^b^	# of act of CAI^b^	Binge drinking^b^	Illicit substance use^b^	Experienced IPV^b^
CSA	*AOR* = 1.5 95% *CI* = 1.1–2.0 *p* = .02	*AOR* = 2.0 95% *CI* = 1.0–1.1 *p* = .10	*b* = 2.0 *SE* = 0.5 *p* = .002	*b* = 4.3 *SE* = 1.6 *p* = .007	*AOR* = 1.6 95% *CI* = 1.2–2.1 *p* = .002	*AOR* = 1.9 95% *CI* = 1.3–2.7 *p* = .001	*AOR* = 1.7 95% *CI* = 1.2–2.3 *p* = .002

^a^ Covariance adjustment for age.

^b^ Covariance adjustment for age and HIV status.

If illicit substance use was examined by individual substance, CSA was significantly associated with increase odds of current use of marijuana (*AOR* = 1.5, 95% *CI* = 1.1–2.1, *p* = .006) and cocaine (*AOR* = 1.5, 95% *CI* = 1.1–2.0, *p* = .01). If IPV was restricted to CTS2 items, CSA was [still] significantly associated with experiencing IPV (*AOR* = 1.6, 95% *CI* = 1.1–2.2, *p* = .007). Separating by type of IPV, CSA was significantly associated with increased odds of currently experiencing physical IPV (*AOR* = 2.0, 95% *CI* = 1.3–3.0, *p* = .002), sexual IPV (*AOR* = 2.5, 95% *CI* = 1.5–4.4, *p* = .001), injurious IPV (*AOR* = 3.0, 95% *CI* = 1.6–5.6, *p* < .001), gay-related IPV (*AOR* = 2.9, 95% *CI* = 1.6–5.2, *p* < .001), and HIV-related IPV (*AOR* = 4.0, 95% *CI* = 1.5–10.5, *p* = .005). Sensitivity analyses included not rectifying potential outliers for number of male sexual partners and number of acts of CAI in the past 90 days; point estimates were unchanged generally beyond the significant figure precision and patterns of significance remained. Thus, conclusions from statistical inferential testing remain unchanged.

For analyses related to the number of syndemic factors experienced by a participant, CSA was significantly associated with the presence of 2 or more syndemic factors for a participant while controlling for age and HIV status (*AOR* = 2.0, 95% *CI* = 1.4–2.9, *p* < .001); being HIV-positive or having an unknown HIV status were also significantly associated with the presence of 2 or more syndemic factors (*AOR* = 1.5, 95% *CI* = 1.1–2.1, *p* = .02; and *AOR* = 3.7, 95% *CI* = 1.9–12.9, *p* = .04 respectively). In fact, participants who met criteria for experiencing CSA were significantly more likely to experience all three syndemic factors—i.e., engagement in HIV risk behaviors, substance misuse, and currently experiencing IPV—while controlling for age and HIV status (*AOR* = 1.6, 95% *CI* = 1.2–2.3, *p =* .006); not knowing one’s HIV status was also significantly associated with the presence of all 3 syndemic factors (*AOR* = 4.5, 95% *CI* = 2.0–9.9, *p <* .001). These results are consistent with CSA being an underlying factor that increases the presence of a syndemic of HIV risk behavior, substance misuse, and IPV among Black MSM and supports having a non-negative HIV status is associated with the presence of the syndemic.

## Discussion

Results indicate that the CSA is prevalent among this large sample of Black MSM and associated with several threats to their well-being during adulthood, specifically HIV, substance misuse, and IPV; in fact, CSA was associated with experiencing multiple problems concurrently. The observed prevalence of CSA in this sample of Black MSM are consistent with concerning, elevated rates among other large studies with MSM—15–37%, some as high as 50% with higher rates using less stringent criteria [18]—and exceed general population prevalence estimates of~20% for women using comparable assessment and ~13% for men [24,25]. The prior multisite study with Black MSM [26] used various indicators/operationalizations for CSA, leading to prevalence rates of 30–51% depending on the criterion; unfortunately, none of the operationalizations used in the prior study are directly comparable to this study. However both these studies found high rates of CSA among the Black MSM.

The prior study found marginal support for the link between CSA and HIV risk indicators among Black MSM in unadjusted and adjusted statistical models (only having >3 male sexual partners in the past 6 months was significantly associated with CSA; most results did not reject the null hypothesis and one for one outcome—having any receptive CAI in the past 6 months—CSA was associated with lower risk). This study found strong support for CSA with increased risk, perhaps lending support for the value and need to include individuals of all HIV statuses (negative, unknown, positive) in assessing CSA and the syndemic among Black MSM. Results from this study lend strong support for CSA being associated with subsequent health, behavioral, and social concerns that are core components of a public health syndemic among MSM, specifically HIV infection, HIV sexual risk behavior(s), substance misuse, and IPV. In fact, results are consistent with CSA being a driving factor for the co-occurrence of HIV sexual risk behaviors, substance misuse, and IPV; i.e., CSA may be a driving factor in the formation and/or presence of a syndemic of among Black MSM.

A major limitation of this study is the reliance on self-reported data; it is possible that those who disclose experiences relating to CSA may be more willing to disclose other sensitive information such as engaging in sexual risk behaviors, using substances, and experiencing IPV. Restricting detection of CSA to those who reported being sexually active may underestimate the exposure to CSA. The study’s definition and assessment of CSA was also restricted to legal dimensions/criteria as well as coded in a binary fashion and, as noted earlier, unable to be directly compared to existing research unfortunately. While the experience of CSA necessarily has preceded the current behaviors and situation among this adult [Black MSM] sample, one cannot infer a direct causal connection, especially since mental health—another key component of the syndemic among MSM—was not assessed in the brief survey. Assessment of substance misuse was limited to any use in the past 90 days, which is a crude measure. The brief survey collected very limited sociodemographic and other contextual information, prohibiting examination or covariance adjustment regarding factors other than age. Finally, since the parent study had a target population of Black MSM couples, some of the participants were in intimate relationships with each other, violating the assumption that observations are independent (e.g., CAI may occur between individuals who are in an intimate relationship with each other) and may be better analyzed using multi-level statistical models; we note that at the screening stage from which these data were collected, respondents did not have to be in a relationship.

The aforementioned limitations do not undermine a central implication from the study findings since self-report and use of legal criteria are likely to result in under-reporting and, thus, under-estimating the prevalence of CSA: there is a pressing need for the prevention and treatment of CSA and its sequelae among Black MSM [of all HIV statuses]. Such efforts would benefit in turn from greater research in several areas: (1) more comprehensive etiological and epidemiological research to gain a more nuanced understanding of CSA as associated dynamics, including factors such as nature/type, relationship to perpetrator(s), age of onset and duration, and disclosure and/or responses to disclosure; (2) intervention research to tailor existing or develop new evidence-based programs that can effectively address the consequences of experiencing CSA, taking into account the unique needs and context of Black MSM; and (3) services research that can better ensure that such programs are not only available to Black MSM, but also increasing the likelihood that they would be willing and able to use such services at all points in the HIV care continuum, from HIV testing for Black MSM who are unaware of their HIV status or HIV-negative, to access and retention in HIV treatment for Black MSM living with HIV.

Given the associations with CSA found in this study, addressing CSA may confer benefits for other public health concerns for Black MSM if indeed CSA is a precipitating event for engagement in subsequent drug and sexual risk behaviors or in the case of IPV, challenging the ability of Black MSM CSA survivors to establishing healthier intimate relationships and/or impeding the ability of them to terminate relationships with partners engaging in risky behaviors. Conversely, interventions for HIV risk reduction [27], compulsive sex (which may foster sexual risk behavior) [11,28,29], and/or substance misuse [11] among Black MSM may also benefit by directly recognizing and addressing CSA.

Advancements in addressing CSA holds promise for addressing a population that shoulders one of the most disproportionate burdens of public health epidemics in the U.S. Not only did CSA precede the observed HIV, substance misuse, and IPV risk indicators and factors, CSA preceded the co-occurrence of these issues. Altogether, this study’s findings are consistent with CSA being an important antecedent—a possible “cornerstone”—of the syndemic experienced by Black MSM in the U.S. Taking this study’s findings together with attendant implications, CSA among Black MSM is not only a fundamental a concern in and of itself, but it is also a matter of social justice and health equity.

## Supporting information

S1 File De-identified data set. (SAV)
